# “Doctors ready to be posted are jobless on the street…” the deployment process and shortage of doctors in Tanzania

**DOI:** 10.1186/s12960-019-0346-8

**Published:** 2019-02-01

**Authors:** Nathanael Sirili, Gasto Frumence, Angwara Kiwara, Mughwira Mwangu, Isabel Goicolea, Anna-Karin Hurtig

**Affiliations:** 1 Department of Epidemiology and Global Health, Department of Public Health and Clinical Medicine, Umeå University, Sweden, 90185 Umeå, SE Sweden; 2 Department of Development Studies, School of Public Health and Social Sciences, Muhimbili University of Health and Allied Sciences, P.O.BOX 65454, Dar es Salaam, Tanzania

**Keywords:** Deployment, Employment of doctors, Rural areas, Internship, Shortage of doctors, Physicians, Health sector, Health workforce, Tanzania, Africa

## Abstract

**Background:**

The World Health Organization advocates that health workforce development is a continuum of three stages of entry, available workforce and exit. However, many studies have focused on addressing the shortage of numbers and the retention of doctors in rural and remote areas. The latter has left the contribution of the entry stage in particularly the deployment process on the shortage of health workforce less understood. This study therefore explored the experiences of medical doctors (MDs) on the deployment process after the internship period in Tanzania’s health sector.

**Methods:**

A qualitative case study that adopted chain referral sampling was used to conduct 20 key informant interviews with MDs who graduated between 2003 and 2009 from two Medical Universities in Tanzania between February and April 2016. These MDs were working in hospitals at different levels and Medical Universities in eight regions and five geo-political zones in the country. Information gathered was analysed using a qualitative content analysis approach.

**Results:**

Experiences on the deployment process fall into three categories. First, “uncertainties around the first appointment” attributed to lack of effective strategies for identification of the pool of available MDs, indecision and limited vacancies for employment in the public sector and private sector and non-transparent and lengthy bureaucratic procedures in offering government employment. Second, “failure to respect individuals’ preferences of work location” which were based on the influence of family ties, fear of the unknown rural environment among urbanized MDs and concern for career prospects. Third, “feelings of insecurity about being placed at a regional and district level” partly due to local government authorities being unprepared to receive and accommodate MDs and territorial protectionism among assistant medical officers.

**Conclusions:**

Experiences of MDs on the deployment process in Tanzania reveal many challenges that need to be addressed for the deployment to contribute better in availability of equitably distributed health workforce in the country. Short-term, mid-term and long-term strategies are needed to address these challenges. These strategies should focus on linking of the internship with the first appointment, work place preferences, defining and supporting career paths to health workers working under the local government authorities, improving the working relationships and team building at the work places and fostering rural attachment to medical students during medical training.

## Introduction

Worldwide, deployment of the health workforce, and in particular medical doctors (MDs), has been among the major challenges facing policy makers for decades [1, 2]. Even in countries where the shortage of MDs is relatively small, the deployment of physicians to rural areas has been a significant challenge [3–6].

**Fig. 1:**
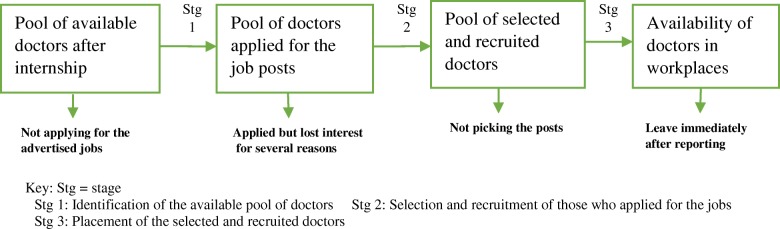
The health workforce deployment stages. Developed by the researchers based on the definition of deployment from the Dictionary of Human Resources & Personnel Management and that of recruitment by Ummuro Adano [7]

According to the *Dictionary of Human Resources and Personnel Management*, deployment means ensuring the availability of the required workforce to perform the assigned tasks at a given time and place. Therefore, deployment of the health workforce is ensuring the availability of the appropriate health workforce to perform the assigned tasks in the health system. The deployment process involves identifying the pool of available staff [numbers, qualifications and available jobs], recruitment of the identified staff and their placement in workplaces (Fig. 1) [7].

Sub-Saharan Africa, home to 11% of the global population and 24% of the global burden of diseases, suffers from a deficit of 0.9 million doctors [8]. The deployment of physicians in sub-Saharan Africa (SSA) is faced with many challenges including the difficulty of attracting, recruiting and placing them in their workplaces [9]. It is estimated that only 25% of the physician workforce serves the rural population, which accounts for 60% of the population in sub-Saharan Africa [10, 11].

### Deployment of doctors in Tanzania

As of 2012, Tanzania’s health sector was operating with less than half the required number health workforce, with a doctor to population ratio of 0.3 per 10 000 individuals nationwide [12].

Post-independence, there have been efforts to address the shortage of health workers to include doctors in the country. Immediate post-independence, the country embarked on task shifting that introduced mid-level non-physician clinicians, the assistant medical officers (AMOs), to cater in places of medical doctors at the district level. The AMOs were trained at an advanced diploma level that prepared them to perform similar roles as the medical doctors at the district level [13]. Although the later was intended to be an interim solution, the availability of doctors at the rural districts where majority of the population resides is still worse [14]. It is documented that only 25% of doctors are in rural Tanzania and thus AMOs have continued to be the backbone of the districts health systems [13].

In early 1990s, Tanzania adopted the ongoing health sector reforms as part of the major socio-economic reforms in the country. The reforms re-introduced the private sector to support the training and deployment of health workers including doctors in the form of public private partnership, and re-introduced a decentralized administration system to strengthen the provision of health services [15, 16]. Following the introduction of decentralized system, the health services provision in Tanzania is organized into a pyramid structure of three levels: the primary level, which contains the district hospital and health facilities below it, the secondary level, which comprises the regional and regional referral hospitals, and the tertiary level, which includes zonal referral, specialized consultant hospitals and the national hospitals. The ministry responsible for health oversees the overall health services provision in all the three levels [17].

In the decentralized health system, districts became the focal points for all health-related programmes’ planning and implementation and thus districts were given mandate to hire and fire health workers among other measures [17, 18]. In 2006, due to many challenges faced the decentralized system [19, 20], another administration system, combining both the decentralized and centralized administration systems named the partial centralized system, was introduced in the health sector [21]. Under the partial centralized system, the deployment of health workers involves a minimum of four government ministries with different roles from need assessment, recruitment, rationalization, validation and approval of new employment posts; and paying salaries to the approved post holders [22].

Despite the introduction of the partial centralized system, shortage and geographical imbalance of health workforce including doctors have persisted [23–25]. The proportion of doctors working in rural areas fell from 31% in 2012 to 26% in 2015 [26]. The partial centralized system is challenged by the small number of approved vacancies compared to the number of graduates, approving vacancies for cadres which are not available on the market, failure to fill the approved vacancies, non-reporting, limited human resources management capacity and limited funding capacity at all levels [26].

The re-introduction of the private sector into the health sector, which was banned in 1977 [15, 27], has witnessed the opening up of private health facilities and training institutions for the health professions [28, 29]. By 2015, the number of medical schools had reached 11 in the country (3 public, 3 private for profit and 5 faith based), 7 out of these medical schools were located in the eastern zone, while the western and southern zones lack a single medical school [30]. Five out of these medical schools had produced at least one cohort of graduates with a doctor of medicine degree and the rest had enrolled medical students [31, 32]. Through the public-private partnership, the government sponsors the training of doctors even for students enrolled into private institutions [31, 33]. The number of MD graduates from all training institutions combined increased from less than 50 in the 1990s to around 1000 annually in 2016 [24, 32]. The training at a medical school is followed by a 1-year mandatory internship programme in hospitals selected by the government [31]. After the internship programme, the doctor is eligible for registration for medical practice through the Medical Council of Tanganyika and for employment in Tanzania [32].

The deployment of MDs in Tanzania is through one of three available routes: (i) apply for employment through the Ministry of Health, in which the doctor will be posted to a district, regional, allied health training institution or any agency under the public sector or seconded to a faith-based organization; (ii) apply for employment in parastatal organizations directly (semi-autonomous hospitals and universities); or (iii) seek employment in the private sector. The commonest route is that of applying through the Ministry of Health. In this route, the Ministry of Health calls for job applications from the doctors whereby one is required to propose three preferred working locations. Following the receipt and analysis of the applications, the ministry makes the final decision for placement according to the approved number of vacancies and the needs across the country.

In order to attract placements to all locations in the country, the government has tried different strategies such as promising priority on career progression to doctors going to rural areas, devising different financial incentives in different locations and coercive posting of doctors [34, 35]. However, their implementation has mostly failed [20, 26] due to scarce resources and weak management capacity to implement the aforementioned strategies [20]. The later has contributed to the persistent shortage and inequitable geographical distribution of doctors in the country [19, 20, 23].

While there is vast literature on the available doctors in numbers and distribution and, to some extent, on the exit issues in Tanzania [24, 32, 36–38], little is documented regarding the deployment process of doctors after the internship period and how it contributes to shortage and uneven geographical distribution of doctors in Tanzania. Anecdotal information shows that the deployment process contributes to shortage and maldistribution of MDs in Tanzania. This study therefore aimed to explore the experiences of MDs on the deployment process after the internship period in Tanzania’s health sector.

## Materials and methods

### Study design

We adopted a case study design in which key informant interviews (KIIs), a qualitative technique, were used for data collection. A case study was used in order to acquire in-depth understanding of the deployment process of MDs in Tanzania. Deployment process of MDs involves many stakeholders in different social and political contexts [39]. Our case study was bounded by the time dimension (MDs graduated between 2003 and 2009), two training institutions (Muhimbili University of Health and Allied Sciences, MUHAS and Kilimanjaro Christian Medical University College, KCMUco) and the level of workplace (from a district to national level and training institutions).

### Study setting

The country is sub-divided into seven geo-political zones (East, West, North, South, Central, Lake and Southern-Highland), 26 regions and 185 districts. The western and southern zones are the zones that suffer most from doctor shortages compared to the rest of the country [24].

This study involved MDs working in district hospitals, regional hospitals, regional referral hospitals, zonal referral hospitals, specialized consultant hospitals, national hospital and medical universities located in 13 districts located in eight regions and five geo-political zones from both the public and private sectors from both urban and rural areas.

### Data collection

We used chain referral sampling [40] to recruit MDs who had graduated from MUHAS and KCMUco between 2003 and 2009. We identified the first two MDs who graduated in 2003 from MUHAS and KCMUco from the graduation books of their respective institutions. Their current workplaces were identified from survey data on the deployment of human resources for health in Tanzania by Muhimbili University through the Swedish International Development Cooperation Agency, which was carried out between 2011 and 2012. From the first two MDs, subsequent MDs were identified and each one helped to locate another one. The chain continued until we attained information saturation. We developed a semi-structured interview guide for carrying out the key informant interviews with the MDs as guided by the three stages of the deployment process (Fig. 1). The questions were constructed based on the existing literature [23, 24, 36, 41] and the experiences of the authors on the shortage and inequitable distribution of MDs in Tanzania.

We conducted the interviews between February and April 2016. We stopped data collection at the 20th interview after we attained information saturation. Out of the 20 participants, 3 were female, 14 were graduates of Muhimbili University and 4 had worked in the private sector for at least 6 months before joining the public sector. The age of all participants ranged between 31 and 42 years. All interviews were conducted in the Swahili language by the first author in the office of the informant and were audio recorded using a digital audio recorder. During the interview process, field notes were taken by the research assistant who accompanied the author. Each interview lasted between 30 min and 1 h.

### Data analysis

Audio-recorded interviews were first transcribed verbatim and then translated from Swahili into English. We analyzed the interviews using qualitative content analysis following Graneheim and Lundman [42]. Qualitative content analysis offers the development of categories from the text data inductively; the inductive derivation of categories is important in capturing experiences from the participants [43].

The full transcripts and field notes were first read and re-read by all authors in order for the researchers to become familiar with the data and the context. Condensed meaning units were then formed through data reduction. These were related to experiences on the deployment of MDs in the health sector in Tanzania. The condensed meaning units were read and re-read in order to extract the codes. Initial codes were discussed and all authors agreed on the revised and final codes. Similar codes were grouped together and through comparison, they were abstracted into sub-categories. Using comparison and the checking and rechecking of similarities and differences between the sub-categories, the sub-categories were sorted to form categories that reflected the manifest content of the interviews.

### Ethical considerations

Muhimbili University of Health and Allied Sciences granted ethical clearance for this study (Ref. No.2015-09-04/AEC/Vol.X/01). Permission to conduct the study was obtained from the Ministry of Health, Community Development, Gender, Elderly and Children (MoHCDGEC), Regional Medical Officers, District Medical Officers, Directors of the hospitals and Heads of the Medical schools. Written informed consent was obtained from each informant before commencing the interview.

## Results

Three categories emerged from the analysis of the experiences of the MDs on the deployment process in the health sector in Tanzania. These were the uncertainties about the first appointment, the failure to respect individuals’ preferences of work location and feelings of insecurity about being placed at a regional and district level.

### Uncertainties about the first appointment

The majority of the informants reported that the period immediately post internship was a period of uncertainties. The few options available during this period were full of uncertainties; these options included waiting for the limited government posts while one was not assured of being selected, or when and where to be posted, seeking a job in the private sector, which has few vacancies and seeking the opportunity for further studies with no clear idea on which area to specialize in and whether s/he will be admitted. One respondent had this to say:

… Maybe about 90% of the doctors are not aware of what they want … Maybe going for a masters’ degree … in which specialization and how. … one might be struggling on where to go and start working … there are those who want to go and earn quick money and those who want to get opportunities for postgraduate training … (KI-06-09-KCMC)

Informants stated that due to the non-transparent and lengthy bureaucratic procedures they were unaware of when posts will be released and how many MDs will be hired. Based on the past experiences of their senior colleagues, it was again clear that not all of the MDs who completed their internship could be employed in the public sector. The number of approved posts was always less than the number of MDs available and the number of posts was increasing slowly compared to the number of MDs graduating annually, which was increasing rapidly. This uncertainty triggered some of them to make a decision on accepting other opportunities that became available even before the release of the government posts.

…the bureaucracy in the employment process contributes to making it look like we have a shortage of doctors but in fact not … .doctors ready to be posted are jobless on the street and there are no jobs. … I have met a lot of colleagues who are ready to be posted anywhere but they are there on the streets, no jobs… (KI-03-09-MUHAS)

Many informants stated that the time taken from announcing job vacancies to the selection and recruitment of successful applicants in the public sector was very long compared to that in the private sector. Although every individual had his/her own experience of waiting, some waited for about 4 months before looking for other opportunities and some waited for more than a year to be selected and posted or to give up waiting for a government post.

…I applied to the ministry immediately after finishing my internship … I was ready to go anywhere and I stated that in the application letter … After waiting for so long I applied for a job in the private sector and within three months I was hired … and that was it, I never turned back (KI-05-09-KCMC)

In order to overcome the aforementioned uncertainties, in some places, planning in advance by the hospital administration made it possible for the MDs to go directly for employment after their internship. Terming the latter as the synchronization of the internship and first appointment; while referring to their own experiences, some informants explained how the synchronization helped them to be employed immediately in their places of first appointment.

…when you are about to finish your internship and you wish to continue working there, you inform the administration that you would like to work there … they ask you to choose which department you would like to work in and they start the recruitment process through the Ministry of Health … immediately you finish your internship you start working there, … that is how I was hired… (KI-01-09-MUHAS)

### Failure to respect individuals’ preferences for work location

The majority of the informants stated that regardless of the given opportunities to propose three work locations, their preferences were not respected. Instead, they were posted to other locations that they had not requested. The reasons given by the majority of informants on making their preferences on where to work included family ties, concern for career prospects and the fear of an unknown rural environment among some urbanized MDs.

The majority of the informants stated that they were compelled by family ties, such as staying close to a family, taking care of sick or aging parents and avoiding foreseeable socio-family consequences like marriage disruptions due to the distance between couples. In general, family ties were an important driver in determining their preference of where to go. When the preference was not offered, the majority of them gave up the government post.

…Ahaaa…I was posted to district *M* but due to family reasons, I did not go… I put much effort on making a follow-up almost daily to know where I had been posted after applying through the district I wanted (*not M*) so as to be close to my family…but surprisingly they posted me to *M*…. Therefore, I decided to seek another job in a nearby place as I had already established my family here… (KI-04-09-KCMC)

While for men the family was a strong factor on working place preference, family ties for women were an even stronger factor. One female informant reported that among other family tie reasons, nurturing her new relationship in view of getting married was an important issue to consider. Therefore, the options she gave for working place was to ensure that she stayed close to her partner. Unfortunately, her given options were not respected and she was posted to a distant region, so she could not go and instead she sought a job in the private sector.

… I applied to be posted to district *H* in Dar es Salaam as I knew there were vacancies … it was this time when I was just approached and my partner was staying in Dar … after repeated follow-ups the ministry posted me to district *T* (located in western Tanzania), I did not go, I opted to seek a job in a private hospital … imagine you are just approached and they post you to another place, it is as if they want to kill that relationship …. (KI-06-09-MUHAS)

The fear of the unknown rural environment among some MDs who had spent all their lives in urban areas was another stated reason to set a preference for which areas they should select for the first appointment. Some stated that this fear was connected to a lack of awareness of the new environment, lack of friends and social groups in the new places compared to their usual places. This fear influenced their decisions on opting for and taking posts in rural or periphery places, and instead they were ready to stay in town areas regardless of whether they were employed in the public or private sectors. This is exemplified by one informant who, when asked whether he would have accepted the first appointment if he was posted to a rural district immediately after internship, said,

…. I would not go just because it is a place which I had never been to before, so I had no established social group there … I was not aware of the living environment there … in short I was not ready to go to a completely new environment or in a place where I knew nobody at all or I have no familiar friends to socialize with … (KI-01-07-KCMC)

### Feelings of insecurity about working at a regional and district level

The feeling of insecurity about working at the regional and district level was expressed by most of the informants. This was attributed to the feeling that many regional and district authorities are not sufficiently prepared to receive and host the MDs, unorganized staff movements at the district and regional hospitals and territorial protectionism among long-serving assistant medical officers.

On explaining their feelings on the local government being unprepared to receive and accommodate the MDs, the majority of the informants stated that the local governments not only lacked financial resources, but it was not part of their planning and therefore often it was not in their budgets. For some of the local authorities, knowing that the MD is paid a salary centrally was enough not to set a budget for housing and other incentives.

… doctors are posted to places where the authorities are not prepared to receive a new person, they may have set a budget for the salary but not the incentives to ensure that this doctor can live and work comfortably. ... We employ a doctor without knowing where s/he is going to live, for instance this person is in Q [one of the remote areas in western Tanzania], how do you ensure a good working environment so as s/he does not think of going back to the city? … (KI-02-05-MUHAS)

Some respondents felt that there was no political will to have MDs at the regions and districts on the part of the local government authorities. The informants believed that the local authorities preferred to manage lower level health staff, hence putting minimum efforts into recruiting MDs in their districts.

… The District Medical Officer and the District Executive Director find it easier to work with these lower cadres as it is easier to manage them compared to those with degrees. … according to the government scheme of service, a medical doctor is a senior government officer and s/he has a higher level of education probably than all the staff in the district … now you become a threat as in some districts even the District Medical Officer were AMO …. (KI-01-03-MUHAS)

In some districts where there was chronic shortage of MDs, the AMOs were appointed to hold the positions of DMOs in charge of the district hospitals or heads of sections. Territorial protection by these AMOs who held the various managerial positions at the districts was mentioned as another cause of feelings of insecurity among the new MDs. Experiences from senior colleagues to the new MDs indicated that these AMOs viewed the incoming MDs as rivals to their positions. This made them struggle in order to secure their positions. The AMOs were either not cooperating with the posted MDs or subjected the new MDs to unfavorable situations like giving them complicated clinical procedures needing vast experience without supporting them. In some places and occasions, these AMOs removed the MDs from the on-call list so that they do not receive the on-call allowances, which is an important financial incentive and sometimes the only financial incentive to many MDs working in the districts.

*…*. I was the only MD, AMOs viewed me as a threat to their positions and they thought that after some time I would be their boss … there was a time they decided to remove me from the list of on-call doctors so that I am not paid the on-call allowances …. (KI-04-03-MUHAS)

In some places where there is a mix of MDs and AMOs, promotion of a teamworking spirit was mentioned to be among the important strategies that have worked in ensuring that AMOs do not see MDs as rivals to their positions but rather colleagues working together. This helped them to be more productive in delivering services to the clients.

… where I was working, we had a good DMO. Immediately when I reported he stated clearly that all workers should respect one another, he told the AMOs that he recognized their competencies and experiences and required them to respect that the MDs had more knowledge than them … for that matter we provided our knowledge and together with their experiences we worked better until when I left for postgraduate training …. (KI-02-09-KCMC)

## Discussion

We explored the experiences of MDs on the deployment process after the internship period in Tanzania’s health sector. We found that the deployment process of MDs in Tanzania’s health sector faces many challenges. Most of these challenges affect the whole terrain of the deployment process starting from identifying the pool of doctors available at the market to the placement process of those who are selected. These findings shed light on the complexity of the deployment process of doctors in Tanzania and give a reflection on its contribution to shortage of doctors in the country.

### Pool of available doctors and employment opportunities

Despite the fact that the capacity of the public health sector to employ MD graduates is far lower compared to the number of MDs who graduates and complete internship annually [24, 32], the private sector is not yet fully developed to accommodate the pool of trained doctors unabsorbed by the government. Lack of an active process to identify the pool of doctors available at the labour market affects the planning process for the training and deployment of doctors in this country. The latter contributes to a lack of reliable statistics on doctors available both in the public and private sectors in this country as revealed by previous studies [24, 36, 37, 44].

In Tanzania where the tracking of graduates is rarely carried out due to many reasons including scarce resources and a weak health information system [45–47], active identification of the pool of doctors available immediately post internship would serve as a better starting point in understanding the magnitude of unemployment in the health sector and for a comprehensive planning process. This is in line with what Sousa et al. [48] documented on the importance of having comprehensive policies in employing the graduating health workers which includes in it understanding the capacity of the health labour market.

### The recruitment process

The influence of family ties on choice of working destination as found in our study conforms to what Araújo et al. [49] documented in 2013 and what Lehmann et al. [50] stated in 2008; job mobility is influenced by family considerations and the influence is greater for women than for men. This is also similar to the findings in a study in Switzerland among female doctors 7 years post-graduation [51], which revealed that most of the female doctors postponed their career growth in favor of taking care of their families and often opted for part-time work.

The experiences of the doctors involved in this study are in line with what Munga in 2009 and the Ministry of Health in 2013 and 2014 wrote on the recruitment process of the health workforce in Tanzania [21, 22, 26]. The latter termed the recruitment of health workers as chaotic, bureaucratic, cumbersome and un-open. The bureaucracy and prolonged waiting time is partly due to the fact that the recruitment of health workers in the public sector in Tanzania involves an interplay of a many stakeholders, at least four government ministries are involved [22].

The chaotic, cumbersome and bureaucratic nature of the employment in public sector contributes to both to non-picking up of first appointment post and non-reporting for those picked up posts in public health facilities and instead join the few available private health facilities [36]. The latter continues to widen the geographical imbalance of the health workforce in the country as most of the few well-established private health facilities are in urban areas.

### The placement process

Although the practice in Tanzania is for the MDs to propose three preferred working destinations when applying for government posts, experiences from this study revealed that these preferences were rarely respected. Failure to respect preferences on working destination might be contributing to what the Ministry of Health documented in 2014 that only 63% of the posted health workers reported to their new workplaces and 13% of them left immediately for several reasons [26].

As revealed by this study, the fear of a new environment among some MDs, who were born, brought up or trained in urban areas made some MDs to feel insecure about going to rural places where they were not familiar with the environment and they had no social groups. This is similar to what Dolea 2009 and 2010 and Roger 2010 have stated [52–54]. The aforementioned studies documented that students with a rural background or who were trained in rural areas are more likely to accept rural posts and stay. This is also in line with experiences from South Africa, where the decision of a health professional to go to practise in rural areas is partly influenced by exposure to rural practice during training, an understanding of rural needs and exposure to rural role models [55]. Although we have not analyzed the origin of students enrolled in medical schools, anecdotally, the majority of students currently in medical schools were born, schooled or raised in town due to the growing urbanization in Tanzania. We therefore feel that one strategy to ensure an understanding of the rural environment is through increasing rural attachments during medical training.

Lehmann in 2008 [50] mentioned career prospects as one of the pull factors for an individual towards a new destination. Our findings point out that although the promise of career support is one of the government incentives to convince MDs to take up posts in periphery regions and districts, its failure to implement it may turn it to be a push factor which will make some doctors avoid those areas where its implementation is not guaranteed. Although some places struggle to implement this strategy, some fail due to limited financial resources and reluctance of the districts to allow the doctors to go in for specialized training, knowing that there is no position for a specialized clinician in the district according to the health sector staffing norm [56–58]. Comparatively, our study found that career support is readily available in large hospitals and in urban areas compared to rural areas.

As revealed by this study, territorial protectionism was a deliberate way of discouraging MDs to stay in their workplace as they were viewed as a threat to the managerial positions of the AMOs in the districts [13, 59]. The territorial protectionism of the AMOs is not unique to Tanzania. Cumbi et al. [60] reported in Mozambique in 2007 that the mid-level providers (técnico de cirurgia) were in conflict with the MDs. This aforementioned study pointed out that despite having worked for a long period in the districts to an extent that they were referred to as the kings of the districts the *técnico de cirurgia* could not recognize the importance of teamwork with the MDs [60]. The authors feel that territorialism between AMOs and MDs can be reduced through the promotion of team work by forming teams that contain the AMOs, MDs and other health workers. Also, mentoring the fresh graduates is important in preparing them to face challenges rather than escaping them. The latter should be done by senior colleagues who work in those districts and regions.

As revealed in this study, unfavorable working conditions at the local government authorities, affect the deployment of doctors in Tanzania. Unfavorable working conditions at the districts and regions in Tanzania were documented by other studies to affect the retention of doctors at the local government level [23, 25, 36, 38, 61]. Furthermore, these findings in line with what Strasser et al. documented in 2016 about access to health care in rural settings in developing countries [62] and what Lehmann et al. documented on health workforce staffing in remote rural areas in middle- and low-income countries [50]. Therefore, this study adds that improving working condition is an important element not only in addressing retention but also the deployment process in order to ensure availability and even geographical distribution of health workforce. The challenges in Tanzania documented in our study and the other aforementioned studies are Lehmann.

Furthermore, health services management style can influence the deployment of doctors. The frequent unorganized staff reallocations across districts and regions as revealed in this study may affect the choice of work place. This is similar to what was documented by Couper [55], on the style of managing the health services in rural workplaces and its influence on the decision of the health workers to accept a post, stay or leave the rural practice.

#### Trustworthiness

To assess the trustworthiness [42] of our study findings, we used the four criteria for assessing qualitative study trustworthiness of credibility, dependability, transferability and conformability as stated by Guba [63]. The credibility of the findings of this study was enhanced through the triangulation of informants from different workplaces, who graduated from different training institutions and who graduated in different years. The dependability of this study was enhanced through the triangulation of the study setting, researchers and carrying out the data collection in the familiar settings of the informants. The categories in this study were inductively generated from the data in order to ensure that they reflect the informants’ perspectives in order to enhance conformability. These were later presented with the support of codes and quotations. Thick description of the study setting, context, data collection process and analysis was used to enhance the transferability of the study findings.

Lastly, it should be taken into account that the findings of this study reflect the situation during the period in which data collection for this study was carried out.

## Conclusion and recommendations

The whole process of the deployment of medical doctors in Tanzania is faced with many challenges that jeopardize the efforts to ensure the availability of adequate and equitably distributed doctors in the country. The deployment process needs to consider all the three stages of identification of the pool available, selection and recruitment of the identified pool and the placement. To address the challenges facing the deployment process in Tanzania, short-term, mid-term and long-term strategies are needed.

Short-term strategies may include synchronization of the internship with the first appointment, careful analysis and respecting preferences of working destination by the ministry responsible for health among the suggested places. In order to avoid non-selection of some places, the ministry should provide a list of areas that the doctors may provide three preferences. The list should identify those areas that are in more critical situation than others. The health services managers across health facilities at all levels should promote teamwork spirit in the workplaces.

Mid-term strategies should focus on creating a harmonized career plan across districts guided by the ministries responsible for health and local governments, and increasing rural attachments during medical training as the current is limited to places nearby the training institutions due to limited financial resources. Training institutions should be supported (both financial and non-financial) by the government and other development partners to increase their capacity for providing rural attachment during training.

Long-term strategies should target addressing the challenges facing the health labour market. This should focus on establishing a comprehensive database of all doctors available and those who will be joining the health sector in Tanzania. The ministry responsible for health should put in place a health workforce development plan that will ensure that adequate number is trained, absorbed and retained. This calls for a comprehensive planning process between the trainers and employers in this country to establish the current and projected needs of medical doctors in the health labour market.

Finally, stakeholders from different sectors including development partners need to come together to address the big question of development to make rural places equally attractive to doctors and other skilled health workers. As our study has primarily dealt with doctors in the public sector, we recommend studies to consider other cadres and include the private sector for a better understanding of the deployment process.
